# The CUL3–KLHL3 E3 ligase complex mutated in Gordon's hypertension syndrome interacts with and ubiquitylates WNK isoforms: disease-causing mutations in KLHL3 and WNK4 disrupt interaction

**DOI:** 10.1042/BJ20121903

**Published:** 2013-03-14

**Authors:** Akihito Ohta, Frances-Rose Schumacher, Youcef Mehellou, Clare Johnson, Axel Knebel, Thomas J. Macartney, Nicola T. Wood, Dario R. Alessi, Thimo Kurz

**Affiliations:** *MRC Protein Phosphorylation Unit, College of Life Sciences, University of Dundee, Dow Street, Dundee DD1 5EH, Scotland, U.K.; †Scottish Institute for Cell Signalling, College of Life Sciences, University of Dundee, Dow Street, Dundee DD1 5EH, Scotland, U.K.

**Keywords:** BTB domain, Cullin–RING E3 ligase (CRL), Kelch-like domain (KLHL domain), Na^+^/Cl^−^ co-transporter (NCC), Na^+^/K^+^/2Cl^−^ co-transporter 2 (NKCC2), SPS1-related proline/alanine-rich kinase/oxidative stress-responsive kinase 1 (SPAK/OSR1), ubiquitin, CUL3, Cullin-3, CRL, Cullin–RING E3 ligase, DCT, distal convoluted tubule, DTT, dithiothreitol, GAPDH, glyceraldehyde-3-phosphate dehydrogenase, GFP, green fluorescent protein, GST, glutathione transferase, HEK, human embryonic kidney, HRP, horseradish peroxidase, KEAP1, Kelch-like ECH-associated protein 1, KLHL3, Kelch-like 3, LC, liquid chromatography, NCC, Na^+^/Cl^−^ co-transporter, NKCC, Na^+^/K^+^/2Cl^−^ co-transporter, NRF2, NF-E2-related factor 2, OSR1, oxidative stress-responsive kinase 1, qRT-PCR, real time quantitative reverse transcription PCR, RBX1, RING-box 1, E3 ubiquitin protein ligase, RPL13A, ribosomal protein L13a, RT, reverse transcription, rTEV, recombinant tobacco etch virus, siRNA, small interfering RNA, SPAK, SPS1-related proline/alanine-rich kinase, TAL, thick ascending limb, TTBS, Tris-buffered saline containing Tween 20, UBE1, ubiquitin-like modifier-activating enzyme 1, UBE2D3, ubiquitin-conjugating enzyme E2 D3, WNK, with no lysine kinase

## Abstract

The WNK (with no lysine kinase)–SPAK (SPS1-related proline/alanine-rich kinase)/OSR1 (oxidative stress-responsive kinase 1) signalling pathway plays an important role in controlling mammalian blood pressure by modulating the activity of ion co-transporters in the kidney. Recent studies have identified Gordon's hypertension syndrome patients with mutations in either CUL3 (Cullin-3) or the BTB protein KLHL3 (Kelch-like 3). CUL3 assembles with BTB proteins to form Cullin–RING E3 ubiquitin ligase complexes. To explore how a CUL3–KLHL3 complex might operate, we immunoprecipitated KLHL3 and found that it associated strongly with WNK isoforms and CUL3, but not with other components of the pathway [SPAK/OSR1 or NCC (Na^+^/Cl^−^ co-transporter)/NKCC1 (Na^+^/K^+^/2Cl^−^ co-transporter 1)]. Strikingly, 13 out of the 15 dominant KLHL3 disease mutations analysed inhibited binding to WNK1 or CUL3. The recombinant wild-type CUL3–KLHL3 E3 ligase complex, but not a disease-causing CUL3–KLHL3[R528H] mutant complex, ubiquitylated WNK1 *in vitro*. Moreover, siRNA (small interfering RNA)-mediated knockdown of CUL3 increased WNK1 protein levels and kinase activity in HeLa cells. We mapped the KLHL3 interaction site in WNK1 to a non-catalytic region (residues 479–667). Interestingly, the equivalent region in WNK4 encompasses residues that are mutated in Gordon's syndrome patients. Strikingly, we found that the Gordon's disease-causing WNK4[E562K] and WNK4[Q565E] mutations, as well as the equivalent mutation in the WNK1[479–667] fragment, abolished the ability to interact with KLHL3. These results suggest that the CUL3–KLHL3 E3 ligase complex regulates blood pressure via its ability to interact with and ubiquitylate WNK isoforms. The findings of the present study also emphasize that the missense mutations in WNK4 that cause Gordon's syndrome strongly inhibit interaction with KLHL3. This could elevate blood pressure by increasing the expression of WNK4 thereby stimulating inappropriate salt retention in the kidney by promoting activation of the NCC/NKCC2 ion co-transporters. The present study reveals how mutations that disrupt the ability of an E3 ligase to interact with and ubiquitylate a critical cellular substrate such as WNK isoforms can trigger a chronic disease such as hypertension.

## INTRODUCTION

Mutations that increase expression of the *WNK* (with no lysine kinase) *1* gene result in an inherited hypertension syndrome termed Gordon's syndrome or PHAII (pseudohypoaldosteronism type II) [1]. Missense mutations in the related *WNK4* gene that alter three close-by non-catalytic residues (Glu^562^, Asp^564^ and Gln^565^) also cause Gordon's syndrome [1]. How these mutations influence WNK4 function is unknown. Most evidence points towards the WNK1 and WNK4 isoforms exerting their effects on blood pressure through their ability to phosphorylate and activate two highly related protein kinases termed SPAK [SPS1-related proline/alanine-rich kinase; also known as STK39 (serine threonine kinase 39)] and OSR1 (oxidative stress-responsive kinase 1) [2–4]. SPAK and OSR1 interact with MO25 (mouse protein-25) isoform subunits to form a maximally activated complex [5]. The SPAK and OSR1 kinases, once activated by WNK kinases, phosphorylate and activate members of the electroneutral cation-coupled chloride co-transporters [SLC12 (solute carrier family 12)], including the NCC (Na^+^/Cl^−^ co-transporter) and NKCC (Na^+^/K^+^/2Cl^−^ co-transporter) 1 and 2, that are targets for the blood-pressure-lowering thiazide diuretic and loop diuretic drugs [4,6–10].

Consistent with the critical role that the WNK1/WNK4-mediated activation of SPAK and OSR1 plays in regulating blood pressure, knockin mice expressing a form of SPAK in which the T-loop residue is changed to alanine to prevent activation by WNK isoforms have low blood pressure and reduced phosphorylation of NCC in the kidney [11,12]. SPAK-knockout mice display a similar phenotype [13]. Patients with Gordon's syndrome are also highly sensitive to thiazide diuretics that target NCC, which is consistent with the WNK signalling pathway regulating these critical ion co-transporters [1]. Previous work has revealed that a significant number of Chinese patients with a low blood pressure condition, termed Gittleman's syndrome, possess a mutation of the major SPAK/OSR1-activating phosphorylation site on NCC (T60M) [4,14].

Exciting recent research has revealed about 50 unrelated familial patients with Gordon's syndrome, possessing no mutations in the WNK isoforms, but instead displaying mutations in either CUL3 (Cullin-3) [15] or the BTB-domain containing protein KLHL3 (Kelch-like 3) [15,16]. CUL3 is the core scaffolding subunit of a subtype of the largest class of E3 ubiquitin ligases in the cell, called CRLs (Cullin–RING E3 ligases) [17,18]. Like all ubiquitin E3s, CRLs transfer ubiquitin from an E2 enzyme on to other proteins, resulting in the formation of ubiquitin chains linked to the substrate. These chains are recognized by a large protease called the 26S proteasome, leading to the proteolytic degradation of the ubiquitin-tagged protein [19]. CUL3 assembles a multi-subunit modular CRL complex by associating with the RING-finger protein RBX1 (RING-box 1, E3 ubiquitin protein ligase) and variable BTB-containing substrate adaptor proteins [20–22]. The BTB domain directly interacts with the Cullin N-terminus, whereas the substrate is recruited through other protein-interaction domains. The best-studied CUL3 substrate adaptor is the Kelch-like protein KEAP1 (Kelch-like ECH-associated protein 1), which regulates the proteasomal degradation of the transcription factor NRF2 {NF-E2-related factor 2; also known as NFE2L2 [nuclear factor (erythroid-derived 2)-like 2]} [23,24]. Structural studies have revealed that NRF2 directly interacts with the Kelch-like domain of KEAP1 to position NRF2 for efficient ubiquitylation by the CUL3–RBX1 core ubiquitin ligase complex [25,26]. Many other BTB proteins that are known to assemble with CUL3 also bind their substrates through a Kelch-like domain [27–29].

The identification of mutations in CUL3 and KLHL3 in Gordon's syndrome patients suggests that these two proteins may also form a CRL E3 complex that regulates blood pressure. The KLHL3 mutations found are either recessive or dominant, whereas CUL3 mutations are dominant. Dominant KLHL3 mutations are clustered in short segments within or nearby the ‘Kelch propeller motif’ or the ‘BTB domain’ [15,16], suggesting that they interfere with either substrate binding or CUL3 binding. All of the CUL3 mutations identified result in skipping of exon 9, producing an in-frame fusion of exon 8 and 10 [15,16]. As with mutations in WNK1 and WNK4, patients with CUL3 and KLHL3 mutations can be effectively treated with thiazide diuretics, which inhibit NCC in the distal nephron of the kidney, suggesting that these mutations activate these ion co-transporters [15,16]. KLHL3 is reportedly also highly expressed in the distal convoluted tubule [15,16].

As a first step in exploring the role that a CUL3–KLHL3 complex may play in regulating blood pressure, we searched for KLHL3-interacting partners. Strikingly, we found that the wild-type KLHL3 immunoprecipitated from mammalian cell extracts indeed interacts with CUL3 and also associates with WNK isoforms, but not other components of the pathway we have tested. We observed that most of the disease-causing mutations in KLHL3 or CUL3 investigated impaired binding to WNK1 or CUL3. We demonstrate that a recombinant wild-type CUL3–KLHL3, but not a disease-causing non-WNK1-binding CUL3–KLHL3 complex, robustly ubiquitylates WNK1 *in vitro* and that siRNA (small interfering RNA) knockdown of CUL3 increases expression of WNK1. We also present evidence that the two missense mutations in WNK4 that cause Gordon's syndrome abolish its ability to interact with KLHL3. The results of the present study demonstrate that the CUL3–KLHL3 complex may regulate blood pressure by interacting with and modulating the ubiquitylation of WNK isoforms. Our findings highlight that disease-causing mutations that disrupt KLHL3–WNK interactions lead to Gordon's syndrome.

## MATERIALS AND METHODS

### Materials

The materials used were: sequencing-grade trypsin (Promega); protease-inhibitor cocktail tablets (Roche); Protein G–Sepharose and all chromatography media (GE Healthcare Lifesciences); anti-GFP (green fluorescent protein)–agarose beads (Chromotek); anti-FLAG M2–agarose, Tween 20, Colloidal Blue staining kit, precast SDS polyacrylamide BisTris gels, Lipofectamine™, blasticidin and hygromycin (Invitrogen); Nonidet P40 (Fluka); ampicillin (Merck); bovine ubiquitin and imidazole (Sigma); and Insect XPRESS™ Media (Lonza).

### Plasmids

The full-length coding region of human KLHL3 (GenBank® accession number NM_017415.2) was amplified by RT (reverse transcription)–PCR from skeletal muscle total RNA (Agilent, catalogue number 540029-41) using the primers 5′-GTAGATCTATGGAGGGTGAAAGTGTCAAGCTG-3′ (forward) and 5′-GTGCGGCCGCTCACAAGGACTTGTGAATCACGGC-3′ (reverse). For mammalian expression this was shuttled into the BamHI/NotI sites of derivatives of pCMV5 and pcDNA5FRT/TO (Invitrogen, catalogue number V6520-20) vectors containing various N-terminal tags. For baculovirus expression the wild-type *KLHL3* gene was re-amplified to add a blunt rTEV (recombinant tobacco etch virus) protease cleavage site to the N-terminus (such that the fusion reads ENLYFQM where M is the initiating methionine residue of KLHL3) and subcloned into the BamHI/NotI sites of a pFBDual vector (Invitrogen, catalogue number 10712-024) modified to include an N-terminal Dac tag [30]. The corresponding KLHL3 R528H mutant construct was obtained by site-directed mutagenesis. Baculovirus co-expression of Dac–Cullin3 and untagged Rbx1 was achieved by subcloning both human Cullin3 (GenBank® accession number NM_003590; synthesized and codon-optimized for insect cell expression by Genscript) and human Rbx1 [GenBank® accession number NM_014248; amplified from IMAGE 3138751 with the primers 5′-GTgctagcATGGCGGCAGCGATGGATGTGGAT-3′ (forward) and 5′-GTggtaccCTAGTGCCCATACTTTTGGAATTC-3′ (reverse), NheI and KpnI sites respectively in lower case] into the BamHI/NotI and NheI/KpnI sites respectively of pFBDual-DAC(TEV). All IMAGE clones (I.M.A.G.E. consortium LLNL) were obtained from Source BioScience U.K. RT–PCR was performed using Superscript III One Step RT–PCR (Invitrogen, catalogue number 12574-018) and PCRs using KOD Hot Start DNA polymerase (Novagen) according to the manufacturers' protocols. Point mutations were introduced using the QuikChange method (Stratagene) in conjunction with KOD polymerase. (RT–)PCR products were subcloned into StrataClone PCR vectors (Agilent, catalogue numbers 240207 and 240205) according to the manufacturer's instructions and fully sequenced prior to further subcloning. DNA sequencing was performed by The Sequencing Service, College of Life Sciences, University of Dundee, Dundee, Scotland (http://www.dnaseq.co.uk).

### General methods

Restriction enzyme digests, DNA ligations, other recombinant DNA procedures, electrophoresis, cell culture, transfections, recombinant protein expression in *Escherichia coli* or insect cells, and protein purification were performed using standard protocols. DNA constructs used for transfection were purified from *E. coli* DH5α cells using Qiagen kits according to the manufacturer's protocol. All DNA constructs were verified by DNA sequencing, which was performed by The Sequencing Service using DYEnamic ET terminator chemistry (GE Healthcare) on Applied Biosystems automated DNA sequencers. WNK1 activity was assessed after its immunoprecipitation by measuring by measuring its ability to phosphorylate kinase-inactive SPAK as described previously [31].

### Antibodies

The antibodies against the following antigens were raised in sheep and affinity-purified on the appropriate antigen by the Division of Signal Transduction Therapy Unit at the University of Dundee: NKCC1 total antibody (residues 1–288 of NKCC1, catalogue number S022D); WNK1 total antibody (residues 2360–2382 of human WNK1, QNFNISNLQKSISNPPGSNLRTT, catalogue number S062B); WNK2 total antibody (residues 1605–1871 of human WNK2, catalogue number S140C); WNK3 total antibody (residues 1142–1461 of human WNK3, catalogue number S156C); SPAK total antibody (full-length human SPAK protein, catalogue number S637B); CUL3 total antibody (residues 554–768 of human CUL3, catalogue number S067D); KLHL3 total antibody (residues 287–587 of human KLHL3, catalogue number S377D); anti-GST (glutathione transferase) antibody (catalogue number S902A); and anti-GFP antibody (catalogue number S268B). The anti-FLAG antibody (F1804) was purchased from Sigma–Aldrich and the anti-GAPDH (glyceraldehyde-3-phosphate dehydrogenase) and anti-RBX antibodies were purchased from Cell Signaling Technology. Secondary antibodies coupled to HRP (horseradish peroxidase) used for immunoblotting were obtained from Pierce.

### Buffers

Buffer A contained 50 mM Tris/HCl (pH 7.5), 0.1 mM EGTA and 1 mM DTT (dithiothreitol). The mammalian cells lysis buffer contained 50 mM Tris/HCl (pH 7.5), 0.15 M NaCl, 1 mM EGTA, 1 mM EDTA, 1 mM Na_3_VO_4_, 50 mM NaF, 5 mM Na_4_P_2_O_7_, 0.27 M sucrose, 1% (w/v) Nonidet P40, 1 mM benzamidine, 0.1 mM PMSF, 0.1% 2-mercaptoethanol and Roche protease inhibitor mix (1 tablet in 50 ml). TTBS (Tris-buffered saline containing Tween 20) was Tris/HCl (pH 7.5), 0.15 M NaCl and 0.2% Tween 20. SDS sample buffer was 1×NuPAGE LDS (lithium dodecyl sulfate) sample buffer (Invitrogen), containing 1% (v/v) 2-mercaptoethanol. The bacterial lysis buffer contained 50 mM Tris/HCl (pH 7.5), 0.15 M NaCl, 1 mM EGTA, 1 mM EDTA, 0.27 M sucrose, 1% (w/v) Triton X-100, 0.1 mM PMSF, 1 mM benzamidine, 0.5 mg/ml lysozome and 0.015 mg/ml DNase containing 1% (v/v) 2-mercaptoethanol.

### Immunoblotting

Samples were heated in sample buffer, subjected to SDS/PAGE and transferred on to nitrocellulose membranes. Membranes were blocked for 30 min in TBST, containing 5% (w/v) dried skimmed milk. The membranes were then incubated overnight at 4°C in TBST containing 5% (w/v) skimmed milk with the indicated primary antibody. Sheep antibodies were used at a concentration of 1–2 μg/ml, whereas commercial antibodies were diluted 1000–2000-fold. The incubation with phosphospecific sheep antibodies was performed with the addition of 10 μg/ml of the dephosphopeptide antigen used to raise the antibody. The blots were then washed three times with TTBS and incubated for 1 h at room temperature (25°C) with secondary HRP-conjugated antibodies diluted 2500-fold in 5% (w/v) skimmed milk in TTBS. After repeating the washing steps, the signal was detected with the enhanced chemiluminescence reagent. Immunoblots were developed using a film automatic processor (SRX-101, Konica Minolta Medical), and films were scanned with at 300-d.p.i. resolution on a scanner (V700 PHOTO, Epson).

### Cell culture, stable cell line generation and transfections

HEK (human embryonic kidney)-293 cells were cultured on 10-cm-diameter dishes in DMEM (Dulbecco's modified Eagle's medium) supplemented with 10% (v/v) foetal bovine serum, 2 mM L-glutamine, 100 units/ml penicillin and 0.1 mg/ml streptomycin. HEK-293 T-REx cell lines stably overexpressing wild-type and mutant forms of KLHL3 and CUL3 were generated using the Flp-in T-REx system from Invitrogen according to the manufacturer's instructions. These human FLAG-tagged wild-type KLHL3, mutant-KLHL3 stable cell lines and the GFP-tagged wild-type KLHL3 were selected/cultured in the presence of 15 μg/ml blasticidin and 0.1 mg/ml hygromycin. Protein expression was induced for 24 h with 1 μg/ml tetracycline. The HEK-293 cells stably expressing NCC were described previously [4]. FLAG-tagged wild-type WNK4 or mutant WNK4 (E562K, Q565E) stably expressed in HEK-293 cell lines using the Flp-in T-REx system were used which have generated in our Unit previously (results not shown). For transient transfection of WNK1 fragments, or other proteins, Lipofectamine™ was used following the manufacturer's instructions. Cells were lysed in 0.3 ml of ice-cold lysis buffer per dish. Lysates were clarified by centrifugation at 26000 ***g*** for 15 min and the supernatants were frozen in aliquots (100 μl) in liquid nitrogen and stored at −80°C. Protein concentrations were determined using the Bradford method. Insect Sf21 cells employed for protein expression were grown in Insect XPRESS™ Media at a density of between 1×10^6^ and 6×10^6^ cells/ml.

### Immunoprecipitations

For immunoprecipitation of FLAG, GST and GFP, FLAG M2–agarose beads, glutathione–Sepharose and anti-GFP–agarose beads were used. For other immunoprecipitations, antibodies were covalently coupled to Protein G–Sepharose employing dimethyl pimelimidate [31], at a ratio of 1 μg of antibody per 1 μl of beads. For immunoprecipitations, 0.2–5 mg of lysates was incubated with 5–10 μl of antibody–resin conjugate for 1 h at 4°C with gentle agitation, and the immunoprecipitates were washed three times with 1 ml of lysis buffer containing 0.5 M NaCl and then twice with 1 ml of Buffer A. Proteins were eluted by resuspending the washed immunoprecipitates in 20–100 μl of 1× SDS sample buffer.

### MS analysis

Lysates (5 mg) derived from HEK-293 cells stably expressing wild-type or mutant FLAG epitope-tagged KLHL3 were subjected to immunoprecipitation with anti-FLAG antibody covalently conjugated to agarose (5 μl). Immunoprecipitates were washed three times with lysis buffer containing 0.5 M NaCl followed by two washes with Buffer A. Proteins were eluted from FLAG beads by resuspending the immunoprecipitates in SDS sample buffer (30 μl). The immunoprecipitates were subjected to electrophoresis on a precast 4–12% gradient gel (Invitrogen) and the protein bands were visualized following Colloidal Blue staining. Proteins in the selected gel bands were reduced and alkylated by the addition of 10 mM DTT, followed by 50 mM iodoacetamide. Identification of proteins was performed by in-gel digestion of the proteins with 5 μg/ml trypsin and subsequent analysis of the tryptic peptides by LC (liquid chromatography)–MS/MS (tandem MS) on a Thermo LTQ-Orbitrap system coupled to a Thermo Easy nano-LC instrument. Excalibur RAW files were converted into peak lists by Raw2msm [32] and then analysed by Mascot (http://www.matrixscience.com), utilizing the SwissProt human database. Two missed cleavages were permitted, the significance threshold was *P*< 0.05.

### Proteins for *in vitro* ubiquitylation reaction

The His–UBE2D3 (ubiquitin-conjugating enzyme E2 D3) E2 conjugating enzyme was expressed in BL21 *E. coli* cells. His–UBE1 (ubiquitin-like modifier-activating enzyme 1; E1) was expressed in insect Sf21 cells. Wild-type KLHL3 or mutant KLHL3[R528H] were expressed with Dac tags [30] in insect Sf21 cells. The CUL3-3–Rbx1 complex was expressed from a multibac vector [33] with a Dac tag on CUL3 and no tag on Rbx1 in Sf21 cells. The tags on UBE2D3, Cul3–Rbx1 as well as wild-type or mutant KLHL3[R528H] proteins were cleaved employing the rTEV protease and the non-tagged proteins were repurified.

### *In vitro* ubiquitylation assays

For each ubiquitylation assay reaction 5 μl of immunoprecipitated endogenous WNK1–protein G–Sepharose conjugate derived from 0.5 mg of HEK-293 cells was used. All other protein components were expressed and purified as described above. The ubiquitylation assays contained 20 mM Hepes/HCl (pH 7.5), 150 mM NaCl, 2 mM DTT, 10% (w/v) glycerol, 8 μM Cul3–Rbx1 complex, 7 μM KLHL3 (wild-type or R528H mutant), 7 μM E1 (UBE1), 60 μM E2 (UBE2D3) and 3000 μM ubiquitin. Reactions were initiated by adding ATP and MgCl_2_ to a final concentration of 1 mM and samples were incubated with agitation at 30°C for 30 min. Ubiquitylation reactions were terminated by the addition of SDS sample buffer and samples were analysed either after separation on a 6% Tris/glycine SDS polyacrylamide gel followed by immunoblot analysis with anti-WNK1 total antibody, or following separation on a precast 4–12% gradient SDS polyacrylamide BisTris gel and immunoblot analysis with anti-KLHL3 total antibody.

### Knockdown experiment of CUL3 in HeLa cells

An siRNA (ON-TARGETplus) oligonucleotide towards human CUL3 was purchased from Thermo Scientific. The target sequence probes used were: Probe 1, 5′-GCACAUGAAGACUAUAGUA-3′ (catalogue number J-010224-09) and Probe 2, 5′-GAAGGAAUGUUUAGGGAUA-3′ (catalogue number J-010224-06). For the scrambled control, negative control siRNA was purchased from Ambion. Transfections were performed as per the manufacturer's instructions, using a concentration of 50 nM of siRNA. At 5 days post-transfection, cells were lysed with lysis buffer, then centrifuged at 26000 ***g*** for 15 min and the supernatants were frozen in aliquots (100 μl) in liquid nitrogen followed by immunoblotting. Quantitative immunoblotting was performed using the LI-COR Odyssey imaging system.

### qRT-PCR (real-time quantitative RT–PCR)

After transfections of CUL3 siRNA oligonucleotides for 5 days in 12-well plates, RNA was isolated using a RNeasy micro kit (Qiagen, catalogue number 74004) and cDNA was made from 1 μg of the isolated RNA using the I-Script cDNA kit (Bio-Rad Laboratories) according to the manufacturer's protocol. qRT-PCR was performed on triplicate samples (10 μl) according to the manufacturer's protocol in a CFX 384 Real time System qRT-PCR machine (Bio-Rad Laboratories). All primers were designed using PerlPrimer and purchased from Invitrogen. The primers used were: WNK1 forward, 5′-CAGCAGGTAGAACAATCCAG-3′ and reverse, 5′-CGTCCCATCAGATAACACAG-3′; GAPDH forward, 5′-TGCACCACCAACTGCTTAGC-3′ and reverse, 5′-GGCATGGACTGTGGTCATGAG-3′; and RPL13A (ribosomal protein L13a) forward, 5′-CCTGGAGGAGAAGAGGAAAGAGA-3′ and reverse, 5′-TTGAGGACCTCTGTGTATTTGTCAA-3′. The primer efficiency was determined and taken into account when evaluating the qRT-PCR data. The data were normalized to the geometrical mean of housekeeping genes, GAPDH and RPL13A. The Pfaffl method was used to analyse the qRT-PCR data in Microsoft Excel and GraphPad Prism.

## RESULTS

### Evidence that overexpressed KLHL3 associates with WNK1

**Figure 1:**
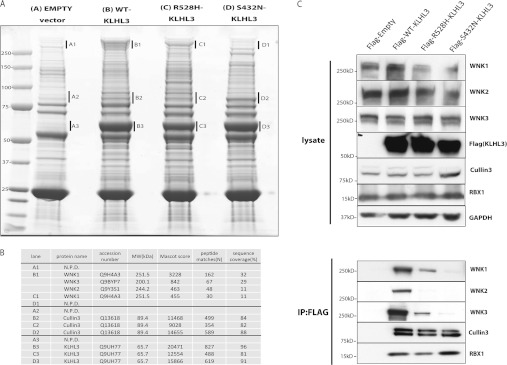
Evidence that KLHL3 associates with WNK isoforms and interaction is impaired by Gordon's syndrome mutations (**A**) Control HEK-293 cells or HEK-293 cells stably expressing the indicated forms of wild-type and mutant KLHL3 possessing an N-terminal FLAG tag were cultured in the presence of 1 μg/ml tetracyclin to induce expression of KLHL3. Cells were lysed and KLHL3 was immunoprecipitated from 5 mg of extract using an anti-FLAG antibody covalently coupled to agarose. The immunoprecipitates were electrophoresed on an SDS polyacrylamide gel and protein bands visualized following Colloidal Blue staining. Molecular masses are shown on the left-hand side in kDa. (**B**) The labelled Colloidal Blue-stained bands identified in (**A**) were excised from the gel and digested with trypsin, and their identities were determined by tryptic peptide mass-spectral fingerprinting, as described in the Materials and methods section. Accession numbers are for the NCBI Entrez Protein database. Mascot protein scores >67 were considered significant (*P*<0.05). N.P.D., no significant protein identity determined. MW, molecular mass. (**C**) As (**A**), except that the immunoprecipitates were immunoblotted with the indicated antibodies. Similar results were obtained in three separate experiments. Molecular masses are shown on the left-hand side in kDa.

To gain insight into how KLHL3 functions, we generated HEK-293 cells that stably overexpress low levels of wild-type KLHL3 or two reported dominant KLHL3 mutations observed in Gordon's syndrome patients (S432N and R528H). We immunoprecipitated KLHL3 and analysed the immunoprecipitations after electrophoresis in a polyacrylamide gel stained with Colloidal Blue (Figure 1A) and Orbitrap MS (Figure 1B). This revealed that in the wild-type and mutant purifications, KLHL3 was indeed the dominant Colloidal Blue-staining band migrating at the expected molecular mass of ~65 kDa, and as expected was not observed in the control immunoprecipitate from HEK-293 cells not overexpressing KLHL3 (Figure 1A). Consistent with CUL3 representing a key binding partner for KLHL3 we observed that CUL3 migrating with a molecular mass of ~90 kDa was co-immunoprecipitated with the wild-type KLHL3 (Figures 1A and 1B), indicating that KLHL3 can form a CRL complex with CUL3. Interestingly, the two selected mutant KLHL3[S432N] and KLHL3[R528H] proteins associated with CUL3 at a similar extent as the wild-type KLHL3, indicating that these mutations do not disrupt the ability to interact with CUL3. The most striking feature of the KLHL3 immunoprecipitations was an intensely Colloidal Blue-staining band migrating at >250 kDa observed with wild-type KLHL3 whose intensity was markedly reduced in the KLHL3[R528H] mutant and was absent from the KLHL3[S432N] mutant (Figure 1A). This band was also absent from the control immunoprecipitate undertaken from non-KLHL3-overexpressing HEK-293 cells. Strikingly, MS analysis revealed that this region of the gel derived from wild-type KLHL3 immunoprecipitate contained significant levels of WNK1, WNK2 and WNK3 isoforms emphasized by very high Mascot scores (WNK1-3228, WNK2-842 and WNK3-463; Figure 1B). WNK1 was also detected within the KLHL3[R528H] mutant immunoprecipitate, but consistent with the reduced intensity of the >250 kDa band observed by Colloidal Blue staining, the Mascot scores for WNK1 was markedly lower (455) than observed for the wild-type KLHL3 (Figure 1B). No WNK2 or WNK3 was detected associated with the KLHL3[R528H] mutant (Figure 1B). In accordance with the absence of the >250 kDa Colloidal Blue-staining band in the KLHL3[S432N] immunoprecipitate, no detectable presence of any WNK isoforms was revealed by MS analysis of these samples.

Immunoblot analysis of KLHL3 immunoprecipitates confirmed observations made by MS that association of the KLHL3[R528H] mutant with WNK1 were significantly reduced compared with the wild-type KLHL3 and that KLHL3[S432N] failed to interact with WNK1 (Figure 1C). This analysis also established that mutant KLHL3[S432N] or KLHL3[R528H] interacted similarly with CUL3 to the wild-type KLHL3.

### Dominant disease-causing KLHL3 mutations interfere with either association to WNK1 or CUL3

**Figure 2:**
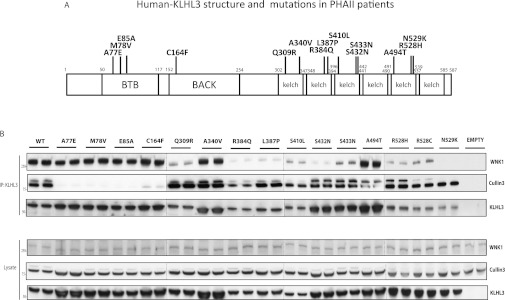
Evidence that dominant KLHL3 Gordon's syndrome mutations impair binding to CUL3 or WNK (**A**) Schematic representation of the domain structure of KLHL3 with the positions of the dominant Gordon's syndrome-associated mutations illustrated. The amino acid boundaries of the domains are indicated. PHAII, pseudohypoaldosteronism type II. (**B**) Control HEK-293 cells or HEK-293 cells stably expressing the indicated forms of wild-type (WT) and mutant KLHL3 possessing an N-terminal FLAG tag were cultured in the presence of 1 μg/ml tetracyclin to induce expression of KLHL3. Cells were lysed and KLHL3 was immunoprecipitated (IP) from 0.2 mg of extract using an anti-FLAG antibody covalently coupled to agarose. The immunoprecipitates (upper panel) as well as whole-cell extracts (lysates, lower panel) were immunoblotted with the indicated antibodies. All gels were immunoblotted in parallel. The broken lines indicate that samples were run on separate gels. Similar results were obtained in three separate experiments. Molecular masses are shown on the left-hand side in kDa.

We next selected 15 different dominant Gordon's syndrome mutations located in different domains of KLHL3 (Figure 2A), generated in stable HEK-293 cell lines, and tested how each mutation affects association with CUL3 and WNK1 (Figure 2B). This strikingly revealed that 13 of the mutations tested either inhibited binding to CUL3 or WNK1, whereas two mutations (A340V and A494T) did not markedly impair association with either protein (Figure 2B). Mutations that impaired binding to CUL3 were located within either the BTB domain (A77E, M78V and E85A) or the adjacent BACK domain (C164F) found in other BTB-containing Kelch proteins which have also been implicated in mediating binding to CUL3 [34]. Interestingly, mutations within the BACK domain of another Kelch-like adaptor protein, KLHL7 (Kelch-like family member 7), cause autosomal-dominant retinitis pigmentosa [35]. Mutations that inhibited KLHL3 binding to WNK1 (Q309R, R384Q, L387P, S410L, S432N, R528H, R528C and N529K) were located in the predicted substrate-binding KLHL repeat motifs.

### Evidence that KLHL3 interacts with WNK1, but not with SPAK/OSR1 or NKCC1/NCC

**Figure 3:**
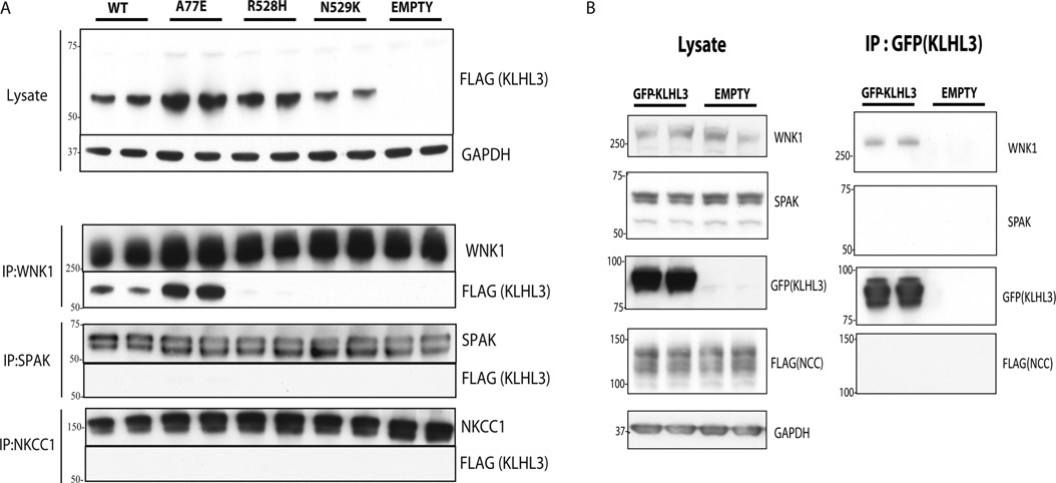
Evidence that wild-type or dominant KLHL3 Gordon's syndrome mutations do not interact with SPAK/OSR1 or NKCC1 or NCC (**A**) HEK-293 stably expressing the wild-type (WT) and indicated mutants of KLHL3 possessing an N-terminal FLAG epitope were cultured in the presence of 1 μg/ml tetracyclin to induce the expression of KLHL3. WNK1, SPAK/OSR1 and NKCC1 were separately immunoprecipitated (IP) from 0.2 mg of extract using previously characterized immunoprecipitating antibodies covalently coupled to Protein G–Sepharose. Whole-cell extracts (lysates, upper panel) as well as immunoprecipitates (lower panels) were immunoblotted with the indicated antibodies. (**B**) N-terminal GFP-tagged wild-type KLHL3 was transiently transfected into previously characterized HEK-293 cells stably expressing FLAG–NCC [4]. At 24 h post-transfection cells were cultured for a further 24 h with 1 μg/ml tetracyclin to induce NCC expression before lysis. GFP–KLHL3 was immunoprecipitated from 0.2 mg of extract employing anti-GFP antibody covalently coupled to agarose. Whole-cell extracts (lysates, left-hand panel) as well as immunoprecipitates (right-hand panel) were immunoblotted with the indicated antibodies. Similar results were obtained in three separate experiments. Molecular masses are shown on the left-hand side in kDa.

To investigate whether KLHL3 might interact with other known components of the WNK signalling pathway we transiently overexpressed wild-type KLHL3 or three disease-causing KLHL3 mutants in HEK-293 cells. We then immunoprecipitated WNK pathway components (WNK1, SPAK/OSR1, NKCC1 and NCC) and tested whether we could detect association of these with KLHL3 by immunoblot analysis. This confirmed association between WNK1 and the wild-type KLHL3, but no detectable co-immunoprecipitation was observed with SPAK/OSR1 (immunoprecipitated with an antibody that recognizes both kinases) or NKCC1 (Figure 3A). We also overexpressed KLHL3 in HEK-293 cells that stably overexpress the NCC ion co-transporter and failed to detect association of KLHL3 with NCC under conditions where interaction with WNK1 was readily observed (Figure 3B).

### WNK1 is a substrate for the CUL3–KLHL3 complex *in vitro*

**Figure 4:**
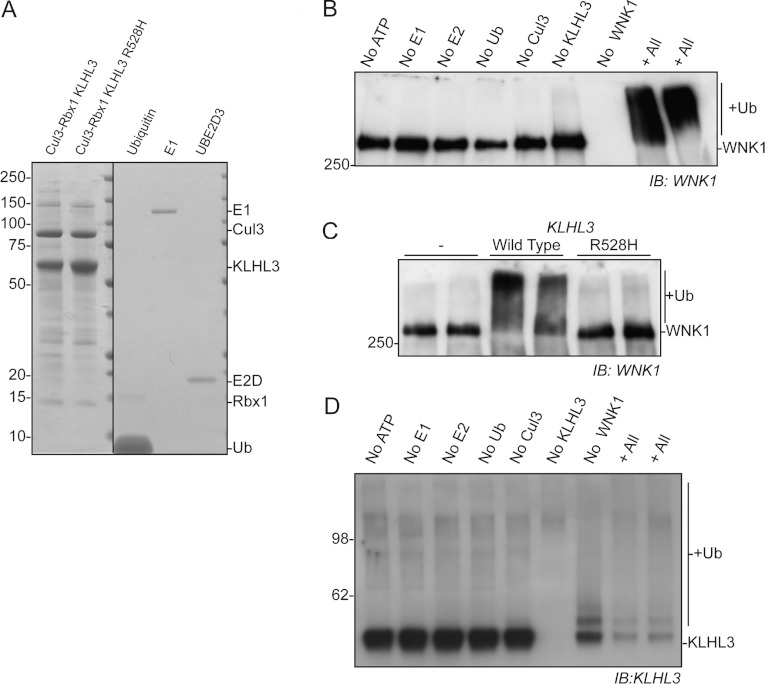
WNK1 is a substrate for the CUL3–KLHL3 complex *in vitro* (**A**) Coomassie Blue-stained SDS/PAGE gel of Cul3–Rbx1–KLHL3 wild-type, Cul3–Rbx1–KHLH3[R528H], ubiquitin (Ub), E1 and UBE2D3 used in the *in vitro* reactions in which all tags used for affinity purification of each protein has been removed with the rTEV protease and the proteins were re-purified. The line indicates that samples were run on separate gels. (**B**) Immunoprecipitated WNK1 from HEK-293 cells was incubated at 30°C for 30 min with purified E1, E2 (UBE2D3), Cullin3–Rbx1, KLHL3 and ubiquitin in the presence of 1 mM ATP. Control reactions were performed whereby one of the proteins required for ubiquitylation was omitted from the reaction as indicated. Samples were subjected to immunoblot (IB) analysis with an anti-WNK1 antibody. (**C**) Ubiquitylation reactions were performed as in (**B**) and the ability of KLHL3 R528H to promote WNK1 ubiquitylation was analysed. Independent duplicate reactions are shown. (**D**) Ubiquitylation reactions were performed as in (**B**) and the ubiquitylation of KLHL3 was analysed by immunoblot detection with anti-KLHL3 total antibody. Similar results were obtained in three separate experiments. Molecular masses are shown on the left-hand side in kDa.

To investigate the ability of the CUL3–KLHL3 complex to directly ubiquitylate WNK1, we expressed and purified the CUL3–RBX1 core complex, as well as the wild-type KLHL3 and mutant KLHL3[R528H] (Figure 4A). Wild-type KLHL3 and CUL3–RBX1 were mixed and used in *in vitro* ubiquitylation reactions that also contained ubiquitin E1, ubiquitin E2 (UBE2D3) and ubiquitin. When immunoprecipitated WNK1 was added to the reaction in the presence of magnesium and ATP, we observed extensive ubiquitylation of WNK1 (Figure 4B). Moreover, we observed no WNK1 ubiquitylation when the WNK1-binding mutant form of KLHL3[R528H] was used in the assay in place of the wild-type protein (Figure 4C). WNK1 ubiquitylation was not detected in the absence of either CUL3 or KLHL3 or any of the proteins that function upstream of the CRL in the ubiquitylation cascade (E1, E2D and ubiquitin; Figure 4B). These results demonstrate that CUL3–KLHL3 forms an active CRL complex that through the binding of KLHL3 to WNK1 can target WNK1 for ubiquitylation. We furthermore detected CUL3–RBX1-dependent ubiquitylation of KLHL3 in the *in vitro* ubiquitylation reactions, indicating that CUL3–RBX1 could directly regulate KLHL3 (Figure 4D). This reaction was more pronounced in the presence of WNK1 and further work is required to establish whether binding of WNK1 to the KLHL3—CUL3 complex stimulated CRL complex activity resulting in increased ubiquitylation of KLHL3.

### siRNA knockdown of CUL3 increases WNK1 expression and activity in HeLa cells

**Figure 5:**
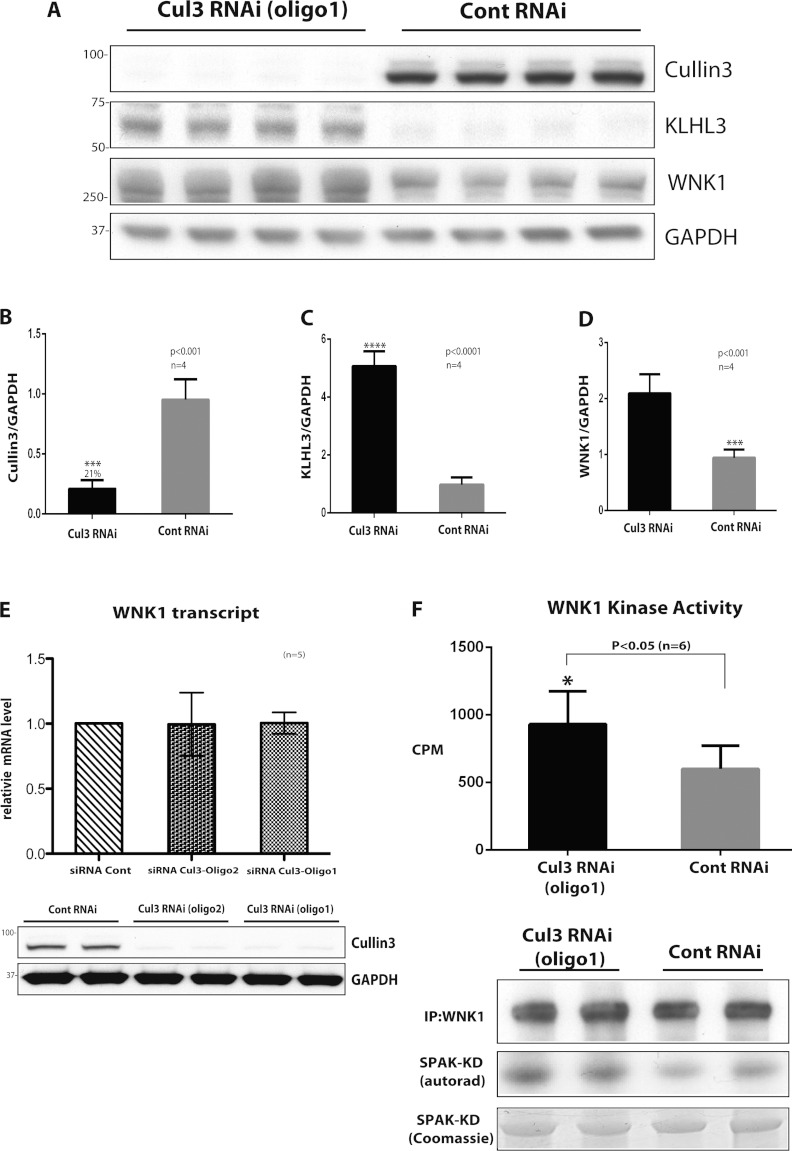
Evidence that CUL3 knockdown enhances WNK1 expression and activity HeLa cells were treated with 50 nM scrambled siRNA or CUL3-directed siRNA (probe 1) for 5 days and cells were lysed. (**A**) Cell lysates were subjected to immunoblotting with the indicated antibody. Each lane contains cell extract from an independent dish of cells from an identical experiment undertaken on the same day. (**B**–**D**) Quantitative LI-COR immunoblot analysis was undertaken and the ratio of CUL3 (**B**), KLHL3 (**C**) and WNK1 (**D**) to GAPDH was quantified. Results are means±S.E.M. for two independent samples each assayed in duplicates with *P* values indicated. (**E**) Total mRNA was isolated from cells and *WNK1* mRNA levels were determined by qRT-PCR. Data were normalized to internal GAPDH and RPL13A controls as described in the Materials and methods section. Results are means±S.D. for three independent samples each assayed in triplicate. (**F**) WNK1 was immunoprecipitated from 0.25 mg of cell extracts and assayed for its ability to phosphorylate kinase inactive SPAK expressed in *E. coli* cells. The top panel displays activity in ^32^P radioactivity incorporated into kinase inactive SPAK (c.p.m.) as mean±S.D. The lower panels display representative immunoblot analysis of WNK1, autorad of phosphorylated SPAK and Coomassie Blue staining of kinase-inactive SPAK employed in the kinase assay. Similar findings were made with an independent CUL3-directed siRNA (probe 2); see data in Supplementary Figure S1 (at http://www.biochemj.org/bj/451/bj4510111add.htm). Cont, control; KD, kinase-dead; IP, immunoprecipitation; RNAi, RNA interference.

We next attempted to reduce CUL3 or KLHL3 expression employing an siRNA approach to establish the impact this had on WNK1 expression and activity. Although we were unable to reduce KLHL3 expression robustly with any siRNA tested (results not shown), we could reduce CUL3 expression over 4-fold by employing two different siRNA oligonucleotides purchased from Thermo Scientific (Figure 5 and Supplementary Figure S1 at http://www.biochemj.org/bj/451/bj4510111add.htm). Consistent with the CUL3-dependent ubiquitylation of KLHL3 we observed *in vitro* (Figure 4D), the knockdown of CUL3 was associated with a marked increase in the expression levels of KLHL3 (Figure 5C and Supplementary Figure S1), further suggesting that CUL3 directly regulates the levels of KLHL3. We also observed that CUL3 knockdown consistently increased endogenous WNK1 expression approximately 2-fold (Figures 5A and 5D, and Supplementary Figure S1). Using qRT-PCR we studied the relative mRNA expression of WNK1 and found that CUL3 knockdown did not impact on *WNK1* mRNA levels (Figure 5E), suggesting that the up-regulation of WNK1 under these conditions is not triggered by an elevation of gene expression. We also measured endogenous WNK1 activity after its immunoprecipitation assessed by its ability to phosphorylate kinase-inactive SPAK expressed in *E. coli* cells [31]. This revealed that CUL3 knockdown was accompanied by a ~1.6-fold increase in WNK1 activity (Figure 5F).

### Evidence that Gordon's syndrome WNK4 missense mutations inhibit binding to KLHL3

**Figure 6:**
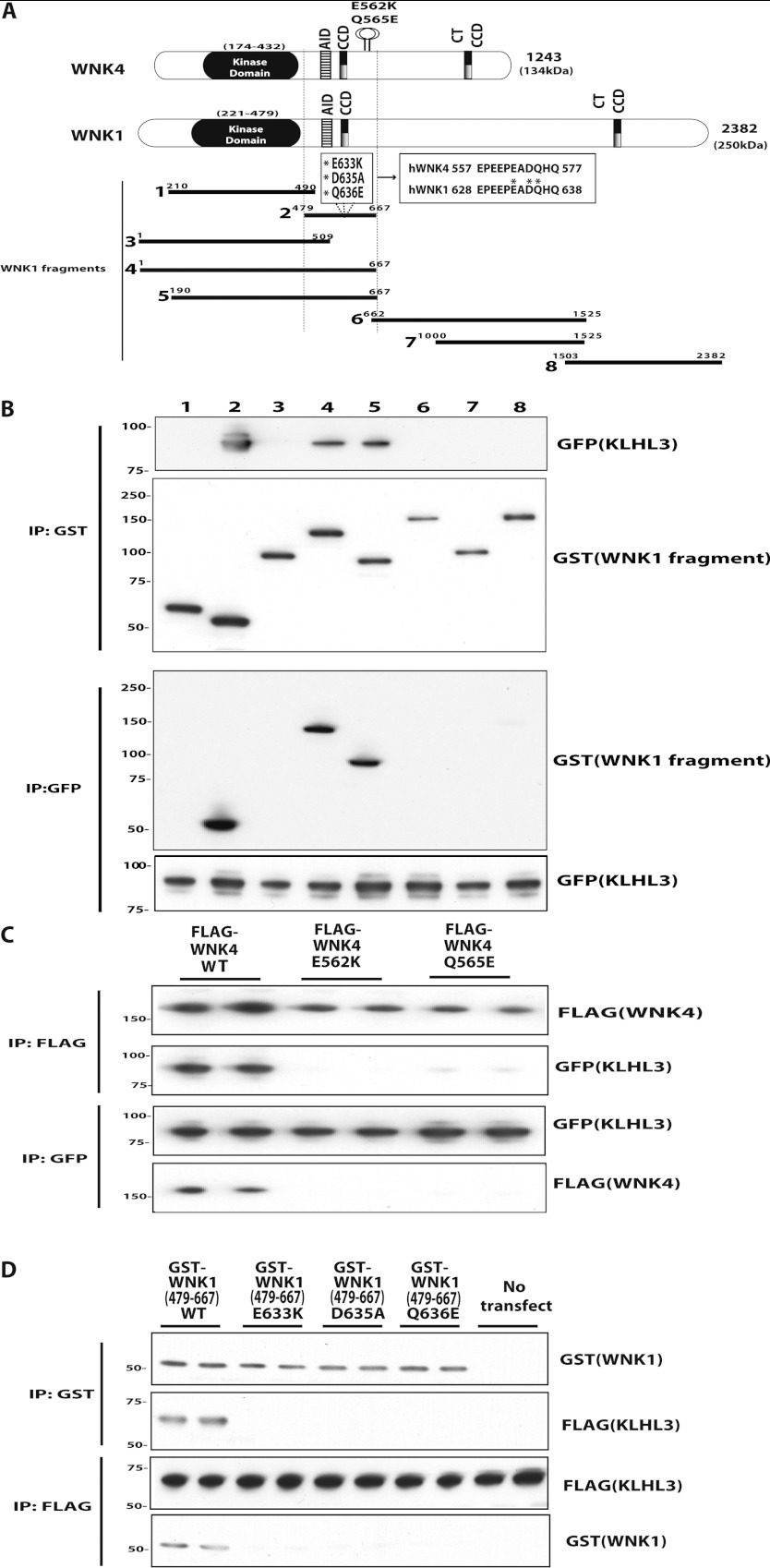
Evidence that WNK4 Gordon's syndrome missense mutations impair binding to KLHL3 (**A**) Schematic diagram of WNK1 and WNK4 isoform structures and the relative placement of their kinase domain, auto-inhibitory domain (AID) and predicted coiled-coiled domains (CCD). Also highlighted in WNK4 is the location of the E562K and Q565E mutations found in Gordon's hypertension patients. CCD, coiled-coil; CT, C-terminal. (**B**) HEK-293 cells stably expressing GFP–KLHL3 were transfected with GST-tagged constructs encoding the indicated fragments of WNK1. At 24 h post-transfection the cells were treated with tetracyclin (1 μg/ml) to induce KLHL3 expression and cells were lysed after a further 24 h. Cell extracts were subjected to affinity purification on glutathione–Sepharose and immunoblotted with an anti-GFP antibody (top panel) or subjected to affinity purification with an anti-GFP antibody and immunoblotted with an anti-GST antibody (upper middle panel). Cell lysates were also subjected to immunoblot analysis with an anti-GST (lower middle panel) or anti-GFP (bottom panel) antibody. Similar results were obtained in two independent experiments. (**C**) HEK-293 cells stably expressing the wild-type (WT) and indicated mutant forms of FLAG-tagged WNK4 were transfected with wild-type GFP-tagged KLHL3. At 24 h post-transfection cells were treated with tetracyclin (1 μg/ml) to induce WNK4 expression and cells were lysed after 24 h. Extracts were subjected to affinity purification using an anti-FLAG antibody (to immunoprecipitate WNK4; upper panels) or an anti-GFP antibody (to immunoprecipitate KLHL3; lower panels) and immunoblotted with the indicated antibodies. Cell lysates were also immunoblotted with the indicated antibodies. Similar results were obtained in two independent experiments. (**D**) As (**B**) except that the wild-type and indicated mutants of the WNK1[479–667] fragment were analysed for their ability to interact with KLHL3. IP, immunoprecipitation. Molecular masses are shown on the left-hand side of the gels in kDa.

To map the region on WNK1 that mediates binding to KLHL3 we expressed various fragments of GST-tagged WNK1 in HEK-293 cells stably expressing the wild-type GFP–KLHL3 and tested which WNK1 fragments interacted. This revealed that the smallest fragment of WNK1 tested that interacted with KLHL3 encompassed residues 479–667, a non-catalytic region immediately C-terminal to the kinase domain that possesses a coiled-coiled domain (Figures 6A and 6B). Interestingly, the equivalent region in WNK4 encompasses residues Glu^562^, Asp^564^ and Gln^565^ located near to the coiled-coiled domain that are mutated in patients with Gordon's hypertension syndrome (Figure 6A). Transgenic and knockin mice expressing these WNK4 mutations display marked hypertension [36,37], but the molecular mechanism by which these mutations disrupt WNK4 function to cause hypertension is unclear. To test whether WNK4 missense mutations affected interactions with KLHL3 we expressed GFP–KLHL3 in HEK-293 cells stably expressing similar levels of the wild-type WNK4 as well as two reported disease-causing mutations (WNK4[E562K] and WNK4[Q565E]). Immunoprecipitation studies confirmed that the wild-type WNK4 robustly bound to KLHL3, but interaction with either disease-causing WNK4 mutant tested was strikingly impaired (Figure 6C). As the residues whose mutation in WNK4 causes Gordon's syndrome are conserved in WNK1 (Figure 6A), we generated the equivalent disease-causing mutations in the KLHL3-binding WNK1[479–667] fragment and observed that all mutations (E633K, D635A and Q636E) ablated binding to KLHL3 (Figure 6D).

## DISCUSSION

The identification of mutations in CUL3 and KLHL3 that cause Gordon's syndrome suggested that this CUL3–KLHL3 ubiquitin ligase complex plays a critical role in regulating blood pressure. We have now demonstrated that CUL3 and KLHL3 indeed interact to form a functional CRL E3 complex. Importantly, we found that in addition to CUL3, KLHL3 also interacts with the WNK1 and WNK4 isoforms mutated in patients with Gordon's syndrome [1]. Moreover, many of the identified Gordon's disease-causing mutations (Q309R, R384Q, L387P, S410L, S432N, R528H, R528C and N529K) located within the putative substrate-binding Kelch-like domain of KLHL3 markedly interfere with binding to WNK1. These data, together with the fact that we were able to successfully reconstitute the KLHL3-dependent ubiquitylation of WNK1 *in vitro* and to increase WNK1 expression following CUL3 knockdown *in vivo*, suggest that WNK1 is a substrate for CUL3–KLHL3 and therefore defines a new substrate adaptor/substrate module for a CUL3 complex implicated in human disease.

The results of the present study furthermore suggest that WNK1 is the critical substrate for CUL3–KLHL3 in the control of blood pressure, as 8 out of 15 mutations that we have tested disrupt the interaction of KLHL3 with WNK1. We have identified five other mutations that interfere with binding to CUL3, which would also be expected to impair the ability of CUL3 to regulate ubiquitylation of WNK1. As expected from these results, a disease-causing mutation of KLHL3 that no longer binds to WNK1 also abolished WNK1 ubiquitylation *in vitro*. As hyperactive WNK1 is known to cause high blood pressure, the control of WNK1 protein abundance by ubiquitylation through CUL3–KLHL3 may be critical for blood pressure control. Thus we propose that a lack, or reduction, of WNK1 ubiquitylation in patients carrying mutations in CUL3 or KLHL3 may result in the observed hypertensive phenotype. Further work is required to investigate this hypothesis.

Given that it is the role of the WNK signalling pathway in the key DCT (distal convoluted tubule) and TAL (thick ascending limb) of the kidney that controls blood pressure, it is possible that regulation of WNK isoforms by the CUL3–KLHL3 occurs mainly in these highly specialized cells geared to regulate salt reabsorption. This might explain why siRNA knockdown of CUL3 in HeLa cells only moderately affected WNK1 expression and possibly more striking effects would be observed in DCT or TAL cells expressing mutated CUL3–KLHL3. Relating to this point it is worth stressing that even a moderate, less than 2-fold, effect on WNK isoform expression in DCT/TAL kidney cells could still have a sufficient impact on WNK signalling to stimulate NCC/NKCC2 inappropriately and induce hypertension. It is likely that further analysis of this system will need to await the availability of Gordon's disease knockin mutations in KLHL3 whose design will be facilitated by the results of the present study. It will be fascinating to establish how mutations that impair KLHL3 binding to WNK isoforms have an impact on WNK isoform protein expression, ubiquitylation and activity and how this affects NCC/NKCC2 ion co-transporters and hence blood pressure.

How missense mutation of three close-by non-catalytic residues on WNK4, namely Glu^562^, Asp^564^ and Gln^565^, which lie immediately after the coiled-coiled domain, lead to Gordon's hypertension syndrome was unknown. In previous work we have not observed any significant change in the intrinsic kinase specific activity of any of the WNK4 disease-causing mutants that we have tested (D. R. Alessi, unpublished work). The finding that disease-causing mutations of Glu^562^ and Gln^565^ markedly impair binding to KLHL3 is very exciting, as this suggests that loss of interaction with CUL3–KLHL3 may lie at the heart of understanding how missense mutations in these residues of WNK4 results in Gordon's syndrome. Interestingly, we have also found that mutating the highly conserved equivalent residues in WNK1 ablates interaction with KLHL3 (Figure 6D). These findings suggest that this region of WNK1 and WNK4 may operate as the major recognition site for the KLHL3–CUL3 complex. In future work it will be important to study levels of WNK4 protein in patient-derived cells (DCT and TAL if feasible) with missense WNK4 mutations of Glu^562^, Asp^564^ and Gln^565^. It would be critical to establish whether WNK4 mutations enhanced the levels of WNK4 protein and how mutations affected the ubiquitylation of WNK4. Although WNK4[D564A] knockin mice that develop hypertension have been described, unfortunately no direct quantitative comparison of the relative expression levels of the wild-type and mutant WNK4 in any tissue was presented in that study [37]. This will be essential to undertake in future work.

It will also be interesting to investigate whether WNK1 ubiquitylation by CUL3–KLHL3 occurs constitutively or in response to a given environmental stimulus. It will be interesting to establish whether binding of KLHL3–CUL3 to the WNK isoforms activates E3 ligase activity. An important question to answer is also whether ubiquitylation of WNK targets it for proteasomal degradation and/or is involved in regulating WNK activity in some other manner. The fact that levels of WNK1 increase after CUL3 knockdown suggests that proteasomal degradation may play some part in WNK regulation. The transcription factor NRF2, the best-studied substrate of CUL3, is constitutively ubiquitylated and targeted for degradation by the ubiquitin–proteasome system and is only stabilized when the cell encounters oxidative stress, which allows it to initiate its protective transcriptional programme [38]. One could similarly imagine a situation where WNK1 is a constitutive substrate of CUL3–KLHL3 and ubiquitylation is only halted upon a hypotonic stimulus to allow salt retention in the kidney. However, the degradation of other CRL substrates is not constitutive, but rather initiated by a signal, which usually results in post-translational modification of the substrate allowing it to be recognized by the CRL [18]. Which of these two possibilities pertains to WNK1 ubiquitylation remains to be determined.

## Online data

Supplementary data
