# Supplementary material for: The CUL3–KLHL3 E3 ligase complex mutated in Gordon's hypertension syndrome interacts with and ubiquitylates WNK isoforms: disease-causing mutations in KLHL3 and WNK4 disrupt interaction
Source: Biochem J. 2013 Mar 14;451(Pt 1):111–22. doi: 10.1042/BJ20121903 (PMC3632089; doi:10.1042/BJ20121903)
Supplement: Supplementary data [file bj4510111add.pdf]

BJ Signal

Biochem. J. (2013) **451**, 111–122 (Printed in Great Britain) doi:10.1042/BJ20121903

online data

SUPPLEMENTARY ONLINE DATA

The CUL3–KLHL3 E3 ligase complex mutated in Gordon's hypertension syndrome interacts with and ubiquitylates WNK isoforms: disease-causing mutations in KLHL3 and WNK4 disrupt interaction

Akihito OHTA*, Frances-Rose SCHUMACHER†, Youcef MEHELLOU*, Clare JOHNSON†, Axel KNEBEL†, Thomas J. MACARTNEY*, Nicola T. WOOD†, Dario R. ALESSI*[1] and Thimo KURZ†[1]

*MRC Protein Phosphorylation Unit, College of Life Sciences, University of Dundee, Dow Street, Dundee DD1 5EH, Scotland, U.K., and †Scottish Institute for Cell Signalling, College of Life Sciences, University of Dundee, Dow Street, Dundee DD1 5EH, Scotland, U.K.

See the following page for Supplementary Figure S1.

Biochemical Journal www.biochemj.org

[1] Correspondence may be addressed to either of these authors (email d.r.alessi@dundee.ac.uk and t.kurz@dundee.ac.uk).

© 2013 The Author(s)
The author(s) has paid for this article to be freely available under the terms of the Creative Commons Attribution Licence (CC-BY)(http://creativecommons.org/licenses/by/3.0/) which permits unrestricted use, distribution and reproduction in any medium, provided the original work is properly cited.

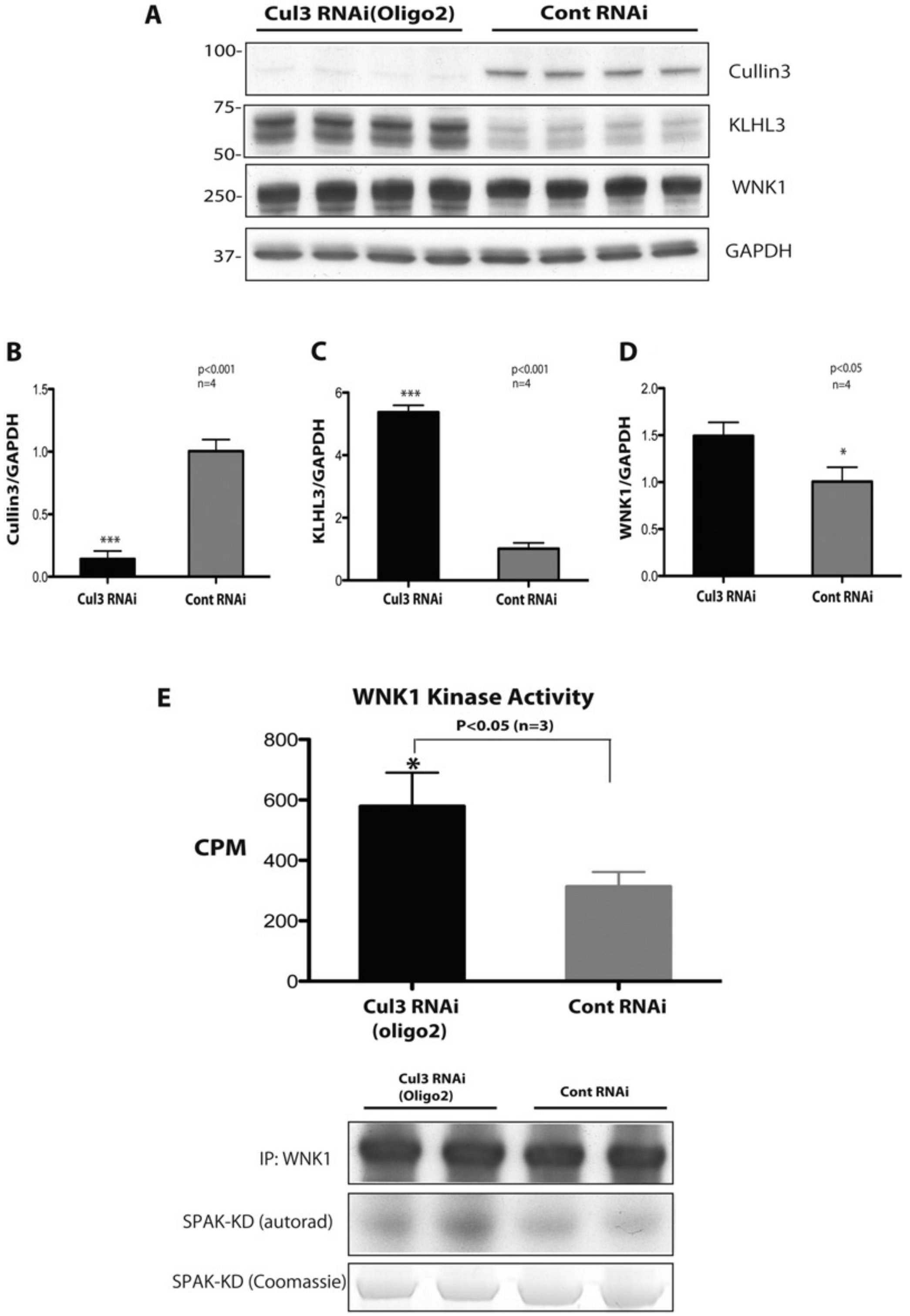

Figure S1 Further evidence that CUL3 knockdown enhances WNK1 expression and activity

HeLa cells were treated with 50 nM scrambled siRNA or CUL3-directed siRNA (probe 2) for 5 days and the cells were lysed. (**A**) Cell lysates were subjected to immunoblotting with the indicated antibody. Each lane contains cell extract from an independent experiment. Molecular masses are shown on the left-hand side in kDa. (**B**–**D**) Quantitative LICOR immunoblot analysis was undertaken and the ratio of CUL3 (**B**), KLHL3 (**C**) and WNK1 (**D**) to GAPDH was quantified. Results are means±S.D. Cont, control; IP, immunoprecipitation; KD, kinase-dead; RNAi, RNA interference.

Received 20 December 2012/4 February 2013; accepted 6 February 2013
Published as BJ Immediate Publication 6 February 2013, doi:10.1042/BJ20121903

© 2013 The Author(s)
The author(s) has paid for this article to be freely available under the terms of the Creative Commons Attribution Licence (CC-BY)(http://creativecommons.org/licenses/by/3.0/) which permits unrestricted use, distribution and reproduction in any medium, provided the original work is properly cited.
